# Insights from Clonal Expansion and HIV Persistence in Perinatal Infections

**DOI:** 10.1128/mBio.00983-21

**Published:** 2021-08-24

**Authors:** Adit Dhummakupt, Deborah Persaud

**Affiliations:** a Johns Hopkins University, School of Medicine, Department of Pediatrics, Division of Infectious Diseases, Baltimore, Maryland, USA

**Keywords:** human immunodeficiency virus, perinatal infections, clonal expansion, latent reservoir

## Abstract

The latent HIV reservoir forms early in the course of infection and is maintained for life despite effective antiretroviral treatment (ART), including early treatment. Perinatal HIV infection presents a unique opportunity to limit seeding of the reservoir through early ART. However, a greater understanding of the persistence of the integrated proviruses is needed for targeting the residual proviruses that form barriers to cure. A study was performed by Bale and Katusiime et al. (M. J. Bale, M. G. Katusiime, D. Wells, X. Wu, et al., mBio 12:e00568-21, 2021, https://doi.org/10.1128/mBio.00568-21) using in-depth integration site analysis in 11 children before ART and after up to nine years of ART. They have identified early development of long-lived proviruses, although the replication competence is unknown. A small fraction of cells bearing integrated proviruses clonally expand early during infection and persist. Integration in the oncogenes *STAT5B* and *BACH2* were also found; these findings confirm the early development of clonal proliferation in perinatal HIV infection despite early effective ART, with a propensity for oncogenes.

## COMMENTARY

Infection with human immunodeficiency virus (HIV) requires lifelong adherence to antiretroviral treatment (ART) in order to maintain an undetectable plasma viral load and immune health. This is because the virus establishes latency in long-lasting memory CD4^+^ T cells (the latent reservoir) that cannot be eliminated with the current therapies, which target replicating virus. Treatment interruption leads to a rapid rebound in plasma viremia with a few notable exceptions (1–5). These reservoir cells have an estimated half-life of 44 months in adults and suggest that elimination of the virus is unlikely within the individual’s life span (6, 7). Further complicating cure strategies, recent seminal studies in children and adults treated during chronic infection have highlighted the ability of a significant fraction (40% in adult infections) of long-lived infected cells to proliferate during ART suppression into expanded clones (8, 9), increasing the pool of replication-competent provirus that is undetectable by the immune system. Clonal expansion of HIV proviruses in the latent reservoir, both defective and intact, is largely from normal immune processes such as antigen-driven stimulation and homeostatic proliferation (10). Although HIV has the ability to integrate into almost any location within the host genome, viral integration sites are enriched in certain regions in both pediatric and adult infection (8, 9, 11). Studies have consistently shown that the provirus is preferentially integrated into active genes (12), possibly due to the ease of integration into a region of open chromatin or proximity to the outer part of the nucleus near the nuclear pore (11). However, following integration events, the provirus is subjected to selective pressure by the immune system resulting in a dynamic proviral landscape in spite of effective ART. One major determinant of the longevity of a given provirus is its ability to remain latent and evade detection from immune surveillance: proviral genomes that are heavily repressed and do not produce viral antigens are less likely to cause cell death or immune clearance. This results in persistence of proviruses in genes that are more likely to be in intergenic regions or in opposite orientation to host genes with long-term ART (13, 14). Furthermore, proviruses integrated into oncogenes such as *STAT5B* and *BACH2* are heavily selected for, possibly through increased cell survival and proliferation (8). The integration into these genes may also favor certain CD4 T cell subsets, such as T regulatory cells (15). Bale and Katusiime et al. (16) have shown that these patterns also appear in the setting of early treated perinatal infection, suggesting that integrated provirus in this region also confers an evolutionary advantage even in the context of an immature immune system. The epigenetic landscape of these infected cells is also drastically different in infants (17), and this may in turn affect integration site and the level of epigenetic repression under ART. Future studies should investigate whether these sites of integration differentially affect T cell subsets, as the T cell population in infants is significantly different from adults and is primarily composed of naive T cells.

The timeline at which the proliferative latent reservoir develops following initial perinatal infection has relevance to strategies to mitigate seeding of the reservoir via initiation of very early ART in infants. Earlier studies have shown that overall, the total proviral reservoir contracts following ART, and the reduction in proviral load follows a biphasic pattern, with a higher rate of decay within 48 weeks following ART initiation (18). However, it has also been shown that there is a paucity of intact provirus that is able to reactivate (14); the defective genomes are not replication competent and are not part of the latent reservoir. Thus, the true decay of the intact reservoir may be obscured by the large proportion of defective genomes that are resistant to clearance. Indeed, the decay dynamics of the intact and replication-competent proviral reservoir has been shown in adults to decay at a faster rate than the defective genomes when assayed with the intact proviral DNA assay (IPDA) (19). In this report by Bale and Katusiime et al. (16), a subset of proviral integration sites was analyzed in comparison to previous single cell sequencing (SGS) to assess clonality, showing a significantly higher level of clonality in the subgenomic sequences from SGS. Determining the cause of this disparity will require the integration site to be coupled with its matching proviral genomic sequence. One strategy has employed a limiting dilution scheme with multiple displacement amplification to enable both integration site analysis and whole-genome sequencing (13), and it can be employed in instances of vertical transmission of HIV.

These studies on the formation of the reservoir in infants will advance our understanding toward HIV remission and functional cure in this unique cohort. Multiple publications have consistently shown that early initiation of ART contributes to lower infant mortality and proviral loads (20–22). One example of the benefits of limiting establishment of the viral reservoir is the Mississippi baby, who was able to achieve remission for over 2 years following discontinuation of ART (1, 23), although the extent to which clonal proliferation led to rebound viremia is the Mississippi baby is not yet known. Other cases have recently been reported of children given early ART, who display virologic control and remission following ART cessation (3, 5). Other studies have detailed the benefits of very early ART initiation for prophylaxis reducing both the total HIV DNA and inducible RNA-producing provirus (24), showing that the seeding of the reservoir with replication-competent provirus is limited by the timing of ART initiation. One critical unanswered question is whether early or very early ART is sufficient in limiting the viral reservoir to the point where a functional cure is possible. As Bale and Katusiime et al. (16) have shown with integration events leading to a long-term persistence, a small proportion of infected cells are able to undergo clonal proliferation to levels that can be detected in peripheral blood cells and are amplified with longer durations of unchecked HIV replication through delayed ART. It is important to note that while clonal populations increase significantly over the course of the study, they still represent a minority of the proviral population; most of the detected integration sites were unique. Although the infants in the CHER trial assessed in this paper initiated ART at between 1.8 and 17 months of age, the infection may have occurred *in utero*, with a reservoir that may be well established before birth and before ART can prevent extensive seeding of the reservoir. Ultimately, more work will be required to determine whether very early ART, such as within the first 48 h of birth, would aid in further reducing reservoir cells capable of clonal proliferation, and whether these proviruses are truly intact and replication competent. Another consideration is the possibility of coinfections with other viruses such as cytomegalovirus (CMV), which has been shown in adults as a mechanism for clonal proliferation that is independent of viral integration into oncogenes (25). This may be especially important in the pediatric population at the age when these coinfections are occurring. Finally, studies on clonal expansion in the context of posttreatment controllers may reveal the relative contributions of this cell population on the inducible reservoir. Recent studies have uncovered clonal proviruses in elite controllers that are present in heavily repressed heterochromatin (26). An examination of clones in perinatally infected posttreatment controllers may reveal mechanisms of repression that prevent the reactivation of this proviral pool. Alternatively, clonal expansion may contribute to eventual rebound viremia in posttreatment controllers. Future studies on determining the significance of clonal expansion of the reservoir in the context of perinatal infections will be crucial to inform curative strategies in this unique population.
